# A decade of clinical trials in Italy (2016–2025): trends, regulatory transformation and biotechnological evolution

**DOI:** 10.3389/fphar.2026.1858688

**Published:** 2026-06-19

**Authors:** Eleonora De Paola, Diego Alejandro Dri, Natalia Maria Verrelli, Fabrizio Galliccia

**Affiliations:** 1 Clinical Trials Office, Italian Medicines Agency (AIFA), Rome, Italy; 2 Department of Pharmacy, School of Specialisation in Hospital Pharmacy, University of Pisa, Pisa, Italy

**Keywords:** AIFA, biotech act, clinical trials, COVID-19, CTIS, EudraCT, OsSC, regulation (EU) 536/2014

## Abstract

**Introduction:**

Italy represents one of major hubs for clinical research in Europe, nevertheless comprehensive long-term analyses of its clinical trial landscape under evolving regulatory frameworks remain limited. This study examines the trends of clinical trials submitted to the Italian Medicines Agency (AIFA) over a 10-year period, evaluating the impact of the COVID-19 pandemic, the transition to Regulation (EU) No. 536/2014 (CTR) and the introduction of the EU Clinical Trials Information System (CTIS).

**Methods:**

All clinical trial applications submitted to AIFA between January 2016 and December 2025 were analysed, integrating data from the Osservatorio sulla Sperimentazione Clinica dei medicinali (OsSC), the European Union Drug Regulating Authorities Clinical Trials Database (EudraCT), and the CTIS. Trials were categorised by year, therapeutic area and Investigational Medicinal Product (IMP) characteristics, and were further analysed by phase, sponsor profile, and study population.

**Results:**

A total of 7,449 clinical trials were submitted over 10 years, showing overall stability with marked fluctuations: a pandemic-driven peak in 2021 (n = 864), a regulatory transition through 2023 (n = 688) and a recovery in 2025 (n = 838). Oncology dominated the therapeutic area distribution, followed by neurological diseases, though both declined; immune-related diseases increased steadily, alongside cardiovascular and respiratory areas. Biological IMPs increased, chemical compounds declined and antibody-drug conjugates tripled by 2025, signaling a biotechnological shift. Phase II and III trials predominated, confirming Italy’s role in confirmatory research. Phase I showed the most consistent growth, especially post-pandemic, while Phase IV decreased. Commercial sponsors dominated and recovered faster post-COVID-19, while non-commercial research declined. The elderly (≥65 years) and paediatric populations remained systematically underrepresented.

**Conclusion:**

The Italian clinical trials setting appears resilient with a tendency toward increased activity, a strong oncology focus, and developments in other areas. It remains highly attractive for industry-sponsored trials yet structurally imbalanced. Persistent weaknesses include limited support for non-commercial research, systematic underrepresentation of key populations and, a decline in independent post-marketing studies. Targeted policy interventions are needed to strengthen academic research capacity and better align clinical research with national and European public health priorities.

## 1 Introduction

Between 2016 and 2025, Italy faced the dual challenge of adapting to a new European regulatory framework and managing the impact of the COVID-19 pandemic. This combined pressure underscores the importance of a systematic 10-year analysis of clinical trial activity to capture long-term structural trends and regulatory cycles.

This decade represents an extraordinary period for clinical research, marked by two major and closely related events:

- The 2016–2025 period was dominated by the anticipation and subsequent implementation of Regulation (EU) No. 536/2014 (CTR) (European Parliament and Council, 2014), with the “go-live” of the Clinical Trials Information System (CTIS) (European Medicines Agency EMA, 2022) on 31 January 2022. The Italian system transitioned from a phase characterized by the fragmentation of Ethics Committees (over 90) and national procedures (2016–2021) to a structural reorganization of the system (reduction to 43 Ethics Committees) and a centralized, coordinated national evaluation process (2022–2025) (Italian Republic, 2018; Italian Republic, 2019; Italian Ministry of Health, 2018; Italian Ministry of Health, 2021; Italian Ministry of Health, 2023a; Italian Ministry of Health, 2023b; Italian Medicines Agency AIFA, 2023; AIFA, 2025a).
- The global COVID-19 pandemic (2020–2022) triggered an unprecedented acceleration in research efforts aimed at identifying effective treatments against SARS-CoV-2, leading to the initiation of the first studies as early as March 2020.

The partial temporal overlap of these events amplified their impact and ultimately accelerated the paradigm shift in clinical trial conduct and oversight. Italy has demonstrated resilience in the transition from the Directive 2001/20/EC (CTD) (Council of the European Union, 2001) to the Clinical Trials Regulation (CTR). Despite a complex and still incomplete national legislative transposition, the Italian Medicines Agency’s (AIFA) activities have allowed trial volumes to remain stable.

Italy does not appear to have experienced major reductions in clinical trial submissions following the introduction of the CTR, unlike several other Member States. According to AIFA’s 2024 activity report, 654 clinical trials were assessed in that year (AIFA, 2025a). Data from the European Medicines Agency (EMA) Key Performance Indicators (KPIs) for 2025 further confirm Italy’s stable contribution to the European clinical trial landscape (Monitoring the European clinical trials environment, 2025a; Monitoring the European clinical trials environment, 2025b; Monitoring the European clinical trials environment, 2025c; Monitoring the European clinical trials environment, 2025d).

In recent years, the Clinical Trials Office (CTO) (Available online ata) has played a central role in ensuring system continuity, despite CTIS operational pressures, demanding deadlines, and technical shortcomings. With an average of only approximately 30 human resources, which has not increased in the meantime, the CTO has managed and continues to ensure the management of a large and rapidly growing number of clinical trial evaluation and authorisation procedures. These procedures increased from more than 3,200 in 2024 to more than 4,500 in 2025, considering all types of trials envisaged by the CTR, in addition to maintaining safety monitoring and standard administrative tasks. Furthermore, the CTO has actively contributed to updating regulatory frameworks, ensuring that institutional processes remain aligned with evolving European and national standards.

Nevertheless, important limitations persist. CTIS does not yet provide a structured channel for direct exchanges between sponsors and Member States, nor does it allow the proposed Ethics Committee (EC) to be encoded as structured data for ethical assessment in line with Article four of the CTR.

At the national level, the incomplete legislative framework for full CTR implementation, combined with the organisational restructuring of AIFA (Italian Ministry of Health, 2024) without a parallel reinforcement of staff and expertise, continues to constrain Italy’s capacity to support sponsors and effectively act as Reporting Member State (RMS). In addition, experience gained after more than 4 years of CTR application has highlighted further issues linked to the Regulation’s own structure.

### 1.1 Global trends in clinical trials

Globally, following the most turbulent years of the pandemic, the biopharmaceutical research and development sector is gradually regaining stability (IQVIA Institute for Human Data Science, 2025). In 2024, the total number of clinical trial starts reached 5,318, returning to the pre-pandemic levels of 2019 (n = 5,316) and slightly exceeding the 2023 count (n = 5,302). However, this quantitative stability is accompanied by profound geographic and thematic shifts.

Despite its strong scientific base, Europe is trailing its competitors regarding advancements in biotechnology. For example, the EU is lagging behind in venture capital investment in health biotech, with a global share of 7%. At the same time, the EU/EEA global share of commercial clinical trials fell from 22% to 12% in just 10 years, while China’s share tripled from 5% to 18% (European Union, 2018; Questions and answers on the european biotech act, 2026). Structural barriers, and complex regulations are prompting innovative European start-ups and scientists to move abroad and ultimately delay patient access to promising new treatments (Questions and answers on the european biotech act, 2026). Currently, around 6,500 advanced-phase trials in the EU are testing medicines that show significant promise for patients (A biotechnology_2025_clinical-trials_en.pdf, 2025).

However, average authorisation times for multinational studies in the EU (approximately 113 days) remain significantly longer than the 60-day average in the U.S. and China, directly impacting time-to-market and patient access to therapeutic innovations (European Union, 2018).

The United States remains the primary hub of attraction, hosting 35% of global clinical trial starts in 2024, followed by China-based companies with a 30% share, though over 80% of these Chinese studies involve exclusively domestic sites. In contrast, Europe’s contribution declined to 21%, confirming a long-term downward trend (IQVIA Institute for Human Data Science, 2025). From the perspective of therapeutic areas, oncology continues to dominate with 41% of clinical trials, but obesity has emerged as the fastest-growing area, with a 77% increase in trials in 2024 compared to 2023 (IQVIA Institute for Human Data Science, 2025). Concurrently, there is a worrying decline in studies on non-COVID infectious diseases, with 176 fewer trials than in 2019, set against the backdrop of the growing threat of antibiotic resistance (IQVIA Institute for Human Data Science, 2025).

In oncology, innovative mechanisms of action are playing an increasingly significant role: Antibody-Drug Conjugates (ADCs), cell and gene therapies, and multispecific antibodies collectively represented 35% of trials started in 2024, while the share of small molecules continues to shrink across all therapeutic areas (IQVIA Institute for Human Data Science, 2025).

To reverse this trajectory, Europe is implementing a biennial strategy defined by the Clinical Trials Coordination Group (CTCG) for the 2026–2027 period (HMA - Heads of Medicines Agencies, 2026), aimed to building a more harmonized, predictable, and attractive environment for international clinical research.

The 2026–2027 strategy also intersects with the Accelerating Clinical Trials in the EU (ACT EU) initiative (HMA-EC-EMA, 2024), a program launched in 2022 by the European Commission, EMA, and national Competent Authorities to create a more integrated, efficient, and patient-oriented research ecosystem.

The Biotech Act proposal in 2025 marks the beginning of a new phase aimed at reclaiming European competitiveness (European Union, 2018).

An additional instrument is the pilot project Facilitating and Accelerating Strategic Clinical Trials in the EU (FAST-EU) (HMA - Heads of Medicines Agencies, 2026), supported by the Heads of Medicines Agencies (HMA) and launched in 2026 with a planned duration of 1 year to facilitate and accelerate the launch of innovative clinical trials across the European Union.

Achieving an 11% increase in clinical trials, as proposed by the European Commission and EMA, could generate economic benefits of approximately €2.4 billion and create around 18,000 jobs. Under a more ambitious scenario (+25%), employment could rise to nearly 80,000 jobs, generating about €9 billion in added value. The most ambitious scenario (+50%) would result in approximately 158,000 additional jobs and €17.9 billion in extra economic value (The economic impact of industry clinical trials across Europe, 2026).

### 1.2 The role of clinical trials for Italy

Over the past decade, clinical trials have driven a therapeutic revolution, particularly through immunotherapies, true blockbuster innovations that have extended survival by years and turned certain cancers, such as melanoma, from fatal diseases into chronic conditions. Likewise, new classes of drugs have transformed the course of metabolic diseases like diabetes and infectious diseases such as hepatitis C, effectively curing around 600,000 Italian patients (Clinical trial day Italia, 2026).

Clinical research thus represents not only a driver of therapeutic innovation but also an engine of economic and social growth for the country, a long-term investment that can significantly contribute to recovery from ongoing healthcare and economic crises by fostering partnerships between the public and private sectors.

### 1.3 Emerging trends and future perspectives

The clinical research ecosystem is rapidly evolving toward more digitalized and patient-centered models. The adoption of Decentralized Clinical Trials (DCTs), the use of Electronic Health Records (EHRs) as primary data sources (eSource), and the integration of Real-World Data (RWD) registries offer concrete opportunities to improve study efficiency and enhance population representativeness. The COVID-19 pandemic acted as a major catalyst for the acceptance and implementation of DCTs, accelerating the adoption of decentralized and digital solutions in clinical research. In this context, Italy has introduced regulatory and operational guidance to support decentralization and digitalization, including the use of eSource, although implementation remains heterogeneous across study sites. In particular, AIFA issued a guideline on regulatory simplification and elements of decentralisation for the conduct of clinical trials of medicines in accordance with Regulation (EU) No. 536/2014, which focuses on organizational aspects specific to the Italian setting (Italian Medicines Agency, 2014).

However, in Italy, the implementation of these innovations is still hindered by regulatory, infrastructural, and cultural barriers.

Another key challenge concerns the financial and organizational sustainability of non-commercial clinical trials, often promoted by academic institutions or Istituti di Ricovero e Cura a Carattere Scientifico (IRCCSs)^1^ which play a crucial role in maintaining research independence.

In this framework, a systematic assessment of the current state of clinical research in Italy is essential to identify the strengths and weaknesses of the system. An integrated approach—considering regulatory, organizational, economic, and qualitative dimensions—is needed to design effective improvement strategies, enhance Italy’s international competitiveness and, ensure patients faster access to innovative therapies.

The present study therefore aims to provide a snapshot of clinical trial trends in Italy during the crucial decade from 2016 to 2025. A 10-year timeframe is sufficiently long to.

- Identify Structural Trends: distinguish random year-to-year fluctuations (e.g., linked to funding availability) from underlying structural dynamics.
- Assess System Resilience: evaluate Italy’s long-term ability to remain attractive and competitive, not merely in response to temporary crises (such as economic downturns or the pandemic) but within a context of evolving regulatory requirements.
- Capture Extraordinary Events and Their Effects: the decade includes disruptive events that can only be properly understood through a broader temporal lens. In relation to COVID-19 (2020–2022), the pandemic led to a temporary surge in COVID-related studies and a slowdown in other research areas. A decade-long analysis is essential to separate emergency-related distortions from structural trends, assessing whether and how the system returned to normalcy or evolved afterward.
- Evaluate Innovation Capacity: a long-term view enables assessment of whether Italy not only participates in clinical research but also attracts and successfully completes studies leading to the availability of innovative medicines.

In conclusion, a 10-year review provides a comprehensive and robust picture of the evolution of the Italian clinical trial landscape, overcoming the limitations of year-by-year evaluations and providing the empirical basis for informed policy and strategic decisions in healthcare and research.

## 2 Materials and methods

### 2.1 Context and objectives

A thorough and systematic review was conducted on clinical trials submitted to AIFA over the 10-year period from 01 January 2016 to 31 December 2025.

The methodological approach followed a series of precise steps to ensure the validity and robustness of the data and observations derived from the review. Data were drawn from clinical trial applications (CTAs) submitted to AIFA, through the OsSC (Available online atb), the EudraCT (Available online atc) and the CTIS platform (Clinical trials information system, 2026) managed by the EMA. These databases collectively cover all trials submitted in Italy and across the EU under successive regulatory frameworks.

The research aims to characterise the evolution of activities within the national context, focusing on trial number and phase, therapeutic distribution, Investigational Medicinal Products (IMPs) involved, sponsor profile and participant population. By situating national dynamics within the broader European research framework, the study seeks to identify areas of competitive strength, highlight gaps and inefficiencies, and explore opportunities for improved regulatory coordination and institutional alignment. The results aim to provide a basis to guide future governance policies and to strengthen the competitiveness of Italian clinical research.

### 2.2 Regulatory data environment

The review relied on data that are partially available online as open-source information on the official AIFA website and on the open-access platforms EU Clinical Trials Register and CTIS.

- Official AIFA website (https://www.aifa.gov.it/sperimentazioni-cliniche) and EU Clinical Trials Register (https://www.clinicaltrialsregister.eu/ctr-search/search) for CTAs submitted under Directive 2001/20/EC, covering the period 01 January 2016–30 January 2023.
- CTIS (https://euclinicaltrials.eu/search-for-clinical-trials/?lang=en) for CTAs submitted under Regulation (EU) No. 536/2014, active from 31 January 2022 onward.

### 2.3 Dataset selection criteria

All studies were included regardless of the outcome of the authorisation process (authorised, authorised with^2^ conditions, lapsed, not authorised, not valid, withdrawn, under evaluation).

Variables collected included:

− Submission date,
− Trial phase classification,
− Sponsor profile (commercial or non-commercial),
− Study population characteristics including vulnerable populations, age groups (0–17 years; 0–17 years, 18–64 years; 0–17 years, 18–64 years, 65+ years; 18–64 years; 18–64 years, 65+ years; 65+ years) and sex inclusion criteria,
− Therapeutic area (Medical Subject Headings (MeSH)-based classification (Medical Subject Headings, 2026)
− IMP category: chemical, biological, Advanced Therapy Medicinal Product (ATMP) and Antibody-Drug Conjugates (ADCs).

Re-submission: According to the law in force, the sponsor was free to re-submit an authorisation request if needed, after a previous withdrawal or rejection of the application. This is referred to as a re-submitted initial application. For this study, in cases of multiple submissions of the same application, only the latest version was considered, ensuring that analyses reflect the current regulatory status of each study.

Transitioned Trials: Between 31 January 2022, and 30 January 2025, sponsors were required to complete the transfer of ongoing clinical trials by submitting a “transitioning clinical trial application” through CTIS. A clinical trial for which a transitioning application had been approved through CTIS was considered a transitioned trial and all provisions of the CTR were applied accordingly (EMA, 2024). For the purposes of this study, transitioned trials were excluded, as they were clinical trials authorised under the CTD and were already included in the data extracted from the OsSC.

IMPD-Q-only Clinical Trial Application: IMPD-Q only applications represent a workaround (EMA, 2025a; EMA, 2023) designed to address CTIS’s lack of functionality for submitting an IMP when the sponsor of a clinical trial is not the product owner of the IMP. IMPD-Q only applications are not expected to be authorised. Given their function, they were included only in the analysis pertaining to IMPs.

Partial Clinical Trial Application: In the case of partial initial submissions (article 11 of the CTR), the submission date was defined as the date on which the sponsor submitted the Part II documentation.

### 2.4 Data reliability and cross-verification

Data within the regulatory systems are entered directly by the sponsor at submission. However, to ensure completeness and internal consistency, additional quality control measures were applied. Cases of missing, inconsistent, or conflicting data were verified through secondary databases, including: AIFA’s internal CTO administrative database, and the EU Clinical Trials Register (Available online atd). This multi-database triangulation enhanced the integrity of the analytical dataset prior to final aggregation.

### 2.5 Data extraction and validation procedures

Data from 01 January 2016 to 30 January 2023, concerning submissions under the CTD, were extracted from the OsSC and complemented with data available through the EudraCT.

Data from 31 January 2022 to 31 December 2025, concerning submissions under the CTR were extracted from CTIS.

Between 31 January 2022 and 30 January 2023, both systems operated in parallel: sponsors could choose to submit new trials under either the CTD via OsSC or the CTR via CTIS. From 31 January 2023 onward, CTIS became the sole mandatory submission platform for all new trials in the EU. To avoid double-counting, each trial was attributed to the platform through which its initial CTA was submitted.

All data were retrieved via Oracle-based queries and integrated with AIFA’s internal database. The data extraction date was 01 January 2026. The extracted datasets were exported to Microsoft® Excel® (Version 2602 Build 16.0.19725.20126–64 bit) for cleaning, transformation, preliminary analysis, and the elaboration of figures, graphics, and linear trendlines.

### 2.6 Data availability and quality statement

The data analysed in this study were retrieved from three distinct platforms with different data architectures, input field structures, and legal bases, necessitating retrospective harmonization. Initial data extractions were systematically processed to ensure consistency across records originating from the OsSC, EudraCT and CTIS databases.

Because the OsSC/EudraCT platform and the CTIS portal employ different data architectures -particularly regarding the classification of IMPs (chemical, biological, ATMPs, and ADCs) a retrospective data harmonization and reclassification process was performed to reconcile structural discrepancies across registries.

Specifically, IMPs were retrospectively down-mapped from the more granular CTIS classification model to the simplified EudraCT categories using product names, substance names, Anatomical Therapeutic Chemical (ATC) codes, product characteristics terminology. Where direct alignment was not available, substance identification and data cleansing rules were applied, including the hierarchical use of international nonproprietary names (INN), official EU substance names, common names reported in Summary of Product Characteristics (SmPCs), Patient Information Leaflet (PIL), IUPAC/systematic names, CAS registry numbers, and sponsor/company codes, to ensure consistent and standardized IMP classification across databases.

All information was provided by study sponsors and may therefore vary in terms of accuracy and completeness. To address these limitations, a comprehensive data cleaning and standardization process was implemented to transform raw data into a uniform format, correct errors, and resolve inconsistencies. These procedures ensured the reliability, accuracy, and internal consistency of the final dataset, which are essential prerequisites for robust and reproducible data analysis.

## 3 Results

### 3.1 Overview

**FIGURE 1:**
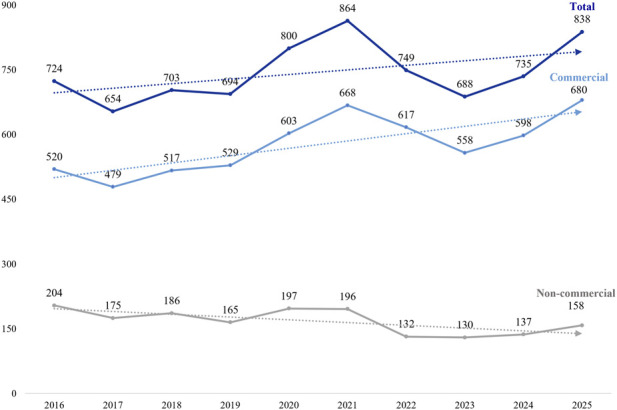
Number of clinical trials (CTs) submitted per year and trends (dotted lines), total and by sponsor profile (commercial vs. non-commercial), 2016–2025.

Between 01 January 2016 and 31 December 2025, a total of n = 7,449 Initial CTAs were submitted to the Italian Medicines Agency (AIFA), with an annual average of approximately 745 studies. This indicates substantial long-term stability within the system, despite significant year-to-year fluctuations (Figure 1). Of these, n = 5,214 (69.99%) were submitted through the OsSC/EudraCT platform, while n = 2,235 (30.01%) were submitted via CTIS. After the three-year period from 2020 to 2022—characterised by a higher number of trials compared to previous years and peaking in 2021 with 864 studies, largely driven by COVID-19-related research—the number of submissions declined in 2023 (n = 688). This decline coincided with the transition to the exclusive use of the CTIS platform and the expiration of the previous CTD. During this period, sponsors likely focused their efforts on adapting to and implementing the CTR, as well as completing the transfer of ongoing clinical trials by submitting transitioning CTAs. The year 2024 marked a period of recovery, initially moderate (n = 735), which was fully confirmed in 2025. In that year, the number of submissions (n = 838) nearly reached the peak observed in 2021, approaching the record levels seen during the pandemic. The 2025 figure represents the second-highest level in the entire series, surpassed only by the 2021 peak. This increase suggests a structured recovery in activity, which may reflect a higher degree of system maturity following the regulatory transition and post-pandemic phases.

#### 3.1.1 Phase

**FIGURE 2:**
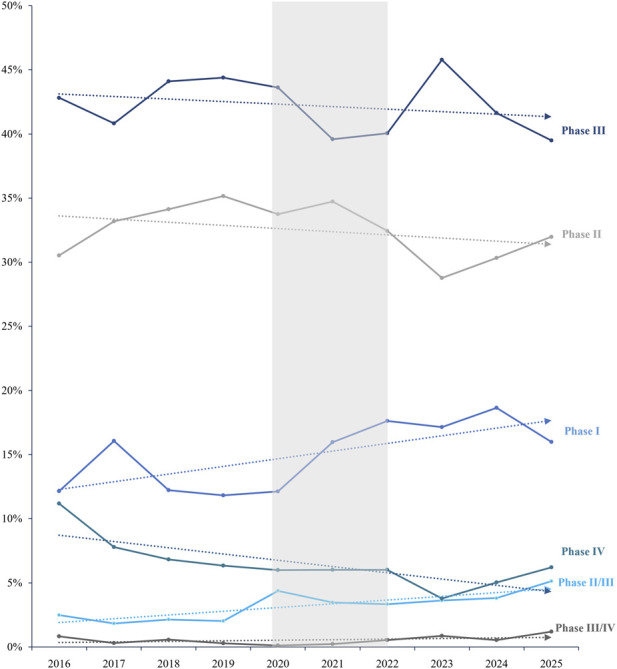
Percentage of clinical trials (% CTs) by phase submitted per year and trends (dotted lines), 2016–2025, the shaded area highlights the 2020–2022 COVID-19 period.

The most common study type was Phase III (n = 3,138/7,449, 42.13%), followed by Phase II (n = 2,424/7,449; 32.54%), Phase I (n = 1,117/7,449; 15.00%), and Phase IV (n = 484/7,449; 6.50%). Integrated Phase II/III trials accounted for n = 245 (245/7,449; 3.29%), and Phase III/IV studies for n = 41 (41/7,449; 0.55%) (Figure 2).

Overall, temporal trends indicate a COVID-19-related disruption during 2020–2022, followed by a decline in 2022–2023 and a partial recovery in 2024–2025, with distinct phase-specific patterns. During the pandemic period, a shift toward earlier-stage research was observed, with Phase I increasing, whereas Phases II and III showed a concomitant reduction. Following a general downturn in 2022–2023 (likely reflecting the post-pandemic reorganization of research activities, particularly visible in Phases II and III), 2025 showed an upward convergence across nearly all active research phases (I, II, and III).

Phase I: Displayed a highly regular and well-defined growth. Starting from 88 studies in 2016 (n = 88/724; 12.15%), it reached 134 studies (n = 134/838; 15.99%) in 2025, with an increase noted after the COVID-19 period. The trend line indicates the most consistent growth among all phases. In contrast to Phases II and III, Phase I shows a continuous rise during the pandemic, increasing from 2020 (n = 97/800; 12.13%) to 2021 (n = 138/864; 15.97%) and further to 2022 (n = 132/749; 17.62%), representing the only phase with continuous growth while Phases II and III declined.

Phase II: It showed solid growth until 2021 (n = 300/864; 34.72%), followed by a sharp decline in 2023 (n = 198/688; 28.78%), before a moderate recovery rebounding in 2025 (n = 268/838; 31.98%), without fully returning to previous peak levels. The reduction began in 2022 (n = 243/749; 32.44%), indicating an earlier downward trend consistent with Phase III.

Phase II/III: Despite low volumes (under 50), it showed a long-term doubling trend, increasing from 18 studies in 2016 (18/724; 2.49%) to 43 in 2025 (43/838; 5.13%).

Phase III: Reached its maximum peak in 2023 (n = 315/688; 45.78%), followed by a gradual decline toward 2025 (n = 331/838; 39.50%), with a relatively stable overall trend over time rather than steady growth. A decrease was already evident in 2021 (n = 342/864; 39.58%) compared to 2020 (n = 349/800; 43.63%), with stabilization in 2022 (n = 300/749; 40.05%).

Phase III/IV: Represents a marginal share of the market, consistently remaining near zero, with a slight increase in 2025 (n = 10/838; 1.19%), though still negligible overall.

Phase IV: This was the only category that exhibited a long-term negative trend. It began 2016 with 81 studies (81/724; 11.19%) and decreased progressively, stabilizing between 40 and 50 in recent years. The lowest value was recorded in 2023 (n = 26/688; 3.78%), possibly reflecting the shift in priorities during and after the pandemic.

#### 3.1.2 Sponsor profile

The dataset revealed a clear imbalance between sponsor categories. Commercial sponsors accounted for 77.45% (n = 5,769/7,449) of all submissions, while non-commercial sponsors—primarily universities and hospital-based research centers—represented 22.55% (n = 1,680/7,449) of the total.

Over time, distinct trends were observed when comparing sponsor profiles following the COVID-19 pandemic (2020–2022) (Figure 1). Both sectors experienced a downturn after 2021 however, the commercial sector recovered much more rapidly, eventually exceeding pre-crisis levels, whereas the non-commercial sector remained significantly below its initial values.

Commercial sponsored trials reached a peak of 668 studies in 2021, followed immediately by a sharp contraction in 2022–2023, dropping to 558 studies in 2023. The recovery began in 2024 (n = 598) and was confirmed in 2025 (n = 680). The overall trend indicates a positive long-term orientation.

The non-commercial sector exhibited an opposite and more restrained pattern. For non-commercial sponsors, the number of studies decreased from 204 in 2016 to 158 in 2025. A moderate recovery occurred 1 year earlier—already in 2023—compared with commercial sponsors.

The lowest point was reached in 2023 with only 130 studies; even though the slight upward trend was confirmed in 2024 and 2025, it was not sufficient to counteract the overall downward trajectory, highlighting a more persistent post-pandemic impact on academic research activity.

Non-commercial sponsors were more frequently involved in Phase IV studies (n = 306/1,680; 18.21%) and, proportionally, also in Phase II (n = 848/1,680; 50.48%), which constituted the largest portion of their activity. In contrast, Phase III trials were predominantly conducted by commercial entities (n = 2,742/5,769; 47.53%), representing nearly half of the entire commercial volume.

These were followed by Phase II (n = 1,576/5,769; 27.32%) and Phase I (n = 1,019/5,769; 17.66%).

**FIGURE 3:**
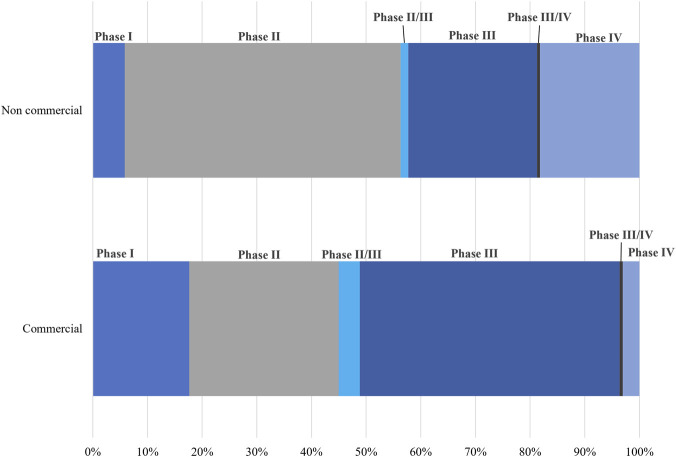
Proportional distribution of clinical trials (% CTs) by phase and sponsor profile (commercial vs. non-commercial), presented as a 100% stacked bar chart, 2016–2025.

Combined phases (II/III and III/IV) represented a minority share for both groups, although the commercial sector clearly led in Phase II/III trials (n = 222/5,769; 3.85%) compared to only 23 (23/1,680; 1.37%) for the non-commercial sector. Phase III/IV trials were marginal for both: 32 commercial (n = 32/5,769; 0.55%) vs. 9 non-commercial (n = 9/1,680; 0.54%). This distribution (Figure 3) reflects the differing objectives of sponsor profiles, with industry focusing on pivotal registration trials, and academic or institutional sponsors more often engaged in exploratory or post-marketing research.

#### 3.1.3 Study population

##### 3.1.3.1 Age

A total of 7,442 clinical trials were retrieved out of 7,449 submitted, 7 clinical trials were excluded due to missing information on the age of the study population. The most significant finding is the extreme concentration of studies on the adult and geriatric populations. The 18–64 years and 65+ years group represented the core of clinical research (n = 5,563/7,442; 74.75%), with a strong prevalence of Phase III studies (n = 2,238/3,136; 71.36%) and reaching nearly 80% in Phase II trials (n = 1,934/2,420; 79.92%) (Table 1).

**TABLE 1:** Number (n) and percentage (n/N, %) of clinical trials (CTs), stratified by age group and study phase.

Age group	Phase I		Phase II		Phase II/III		Phase III		Phase III/IV		Phase IV		Total	
	n	%	n	%	n	%	n	%	n	%	n	%	n	%
0–17 years	63	5.64	137	5.66	29	11.84	306	9.76	1	2.44	29	6.00	565	7.59
0–17 years, 18–64 years	77	6.89	86	3.55	12	4.90	152	4.85	2	4.88	8	1.66	337	4.53
0–17 years, 18–64 years, 65+ years	35	3.13	69	2.85	28	11.43	276	8.80	2	4.88	18	3.73	428	5.75
0–17 years, 65+ years	-	-	-	-	-	-	1	0.03	-	-	-	-	1	0.01
18–64 years	84	7.52	170	7.02	8	3.27	141	4.50	6	14.63	74	15.32	483	6.49
18–64 years, 65+ years	858	76.81	1,934	79.92	168	68.57	2,238	71.36	28	68.29	337	69.77	5,563	74.75
65+ years	-	0.00	24	0.99	-	0.00	22	0.70	2	4.88	17	3.52	65	0.87
N. Total	1,117	100	2,420	100	245	100	3,136	100	41	100	483	100	7,442	100

Studies involving minors, either exclusively or partially, showed significantly lower volumes: the 0–17 years group accounted for 7.59% of the total (n = 565/7,442). Integrated Phase II/III trials and Phase III trials were the categories that enrolled the highest percentages of the 0–17 years population (n = 29/245; 11.84%, and n = 306/3,136; 9.76%, respectively). Studies including both minors and adults (0–17 years and 18–64 years) made up 4.53% of the total (n = 337/7,442). Studies focused exclusively on the geriatric segment (65+ years) are very rare, representing only 0.87% (n = 65/7,442). Only a single protocol in 10 years (n = 1/7,442; 0.01%) included a population of both 0–17 years and 65+ years.

Non-commercial sponsors tended to focus more on adult and geriatric populations (n = 1,276/1,675; 76.18%) compared to industry-sponsored trials (n = 4,287/5,767; 74.34%), whereas commercial sponsors demonstrated a greater inclination to include paediatric participants (n = 477/5,767; 8.27%) versus non-commercial sponsored trials (n = 88/1,675; 5.25%) (Table 2).

**TABLE 2:** Number (n) and percentage (n/N, %) of clinical trials (CTs), stratified by age group and sponsor profile (commercial vs. non-commercial).

Age group	Commercial		Non-commercial		Total	
	n	%	n	%	n	%
0–17 years	477	8.27	88	5.25	565	7.59
0–17 years, 18–64 years	262	4.54	75	4.48	337	4.53
0–17 years, 18–64 years, 65+ years	401	6.95	27	1.61	428	5.75
0–17 years, 65+ years	1	0.02	-	​	1	0.01
18–64 years	328	5.69	155	9.25	483	6.49
18–64 years, 65+ years	4,287	74.34	1,276	76.18	5,563	74.75
65+ years	11	0.19	54	3.22	65	0.87
N Total*	5,767	100,00	1,675	100,00	7,442	100,00

There is a small but distinct difference in research dedicated solely to the elderly: the non-commercial sector showed a proportionally higher interest in studies focused exclusively on those over 65 (n = 54/1,675; 3.22%) compared to the commercial sector (n = 11/5,767; 0.19%).

##### 3.1.3.2 Sex

A total of 7,444 clinical trials were retrieved out of 7,449 submitted, five clinical trials were excluded as they lacked information regarding the sex of the study population.

Regarding sex distribution, participation between males and females was generally balanced with the vast majority of the population enrolled in mixed-sex (“female-male”) studies (n = 6,823/7,444; 91.66%). Conversely, single-sex trials were rare, with those enrolling only men (n = 276/7,444; 3.7%) or enrolling only women (n = 345/7,444; 4.6%).

The commercial sector represented the largest share of the sample (n = 5,768/7,444; 77.49%), while the non-commercial sector accounted for 22.51% (n = 1,676/7,444). Notably, the non-commercial sector demonstrated a significantly higher prioritization of sex-specific research. Specifically, the proportion of studies focused exclusively on the female population in the non-commercial sector (n = 151/1,676; 9.01%) was nearly triple that of the commercial sector (n = 194/5,768; 3.36%). A similar, though less pronounced, trend was observed for exclusively male trials, which were more frequent in the non-commercial sector (4.42% vs. 3.50%). These findings suggest that non-commercial entities may play a more pivotal role in addressing sex-specific health gaps than their commercial counterparts.

##### 3.1.3.3 Vulnerable populations

The majority of the studies (n = 5,995/7,449; 80.48%) included selection criteria involving at least one group considered vulnerable according to the CTD and the CTR:

- subjects who cannot give their express informed consent (n = 1,568/7,449), comprising both minors (n = 1,248) and incapacitated subjects (n = 320).
- pregnant women (n = 71/7,449).
- nursing women (n = 44/7,449).

**FIGURE 4:**
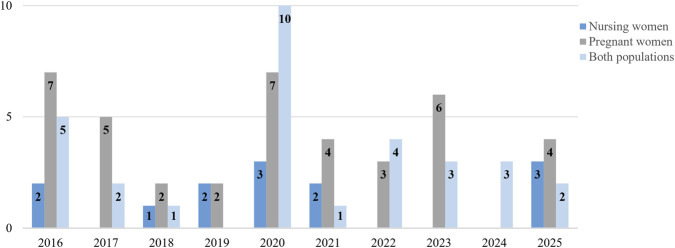
Number of clinical trials (CTs) per year involving pregnant women, nursing women, or both populations, 2016–2025.

Clinical trials enrolling both pregnant or nursing women remained extremely limited during the 2016–2025 period (Figure 4). A distinct peak of interest was recorded in 2020, the first year of the COVID-19 pandemic, when 17 trials included pregnant women, 10 of which also involved nursing women and three exclusively nursing women. This pattern likely reflects the urgent need to generate safety and efficacy data on therapeutic interventions for particularly sensitive populations, such as pregnant women, within an emergency health context. In 2017 and 2019, no nursing women were included in the studies.

Particular consideration must be given to the category of Women of Childbearing Potential (WCBP), regardless of contraceptive use, as these are also classified as vulnerable. Nearly all studies (n = 5,239/7,449; 70.33%) identified women using contraceptives as part of a vulnerable population, either exclusively (n = 5,026/7,449; 67.47%) or in combination with WCBP not using contraceptives (n = 213/7,449; 2.86%). A smaller fraction (n = 245/7,449; 3.29%) included WCBP not using contraceptives. Additionally, 1,965 clinical trials (n = 1,965/7,449; 26.38%) did not report specific information regarding WOCBP. Within this subgroup, 131 trials enrolled exclusively female participants, while the majority (n = 1,554/7,449; 20.72%) included both female and male subjects.

During the 2016–2025 period, a total of 320 clinical trials included incapacitated subjects. Of these: 235 studies (73.43%) were commercial, and 85 studies (26.57%) were non-commercial (Table 3).

**TABLE 3:** Number of clinical trials (CTs) involving incapacitated subjects and subjects in emergency situations, stratified by sponsor profile (commercial vs. non-commercial), 2016–2025.

Year	Incapacitated subjects			Subjects in emergency situations		
	Commercial	Non-commercial	Total	Commercial	Non-commercial	Total
2016	19	11	30	7	7	14
2017	15	7	22	6	5	11
2018	26	10	36	2	12	14
2019	25	6	31	7	4	11
2020	31	17	48	16	21	37
2021	31	14	45	8	10	18
2022	31	7	38	3	4	7
2023	12	6	18	4	6	10
2024	16	4	20	4	2	6
2025	29	3	32	2	3	5
Total	235	85	320	59	74	133

This data highlights a clear prevalence of commercial studies in this field, with a ratio of approximately 3:1 compared to non-commercial trials. This dynamic indicates a progressive difficulty for the non-commercial sector in promoting studies involving incapacitated subjects, likely linked to the greater organizational and regulatory complexity of such trials.

The number of trials enrolling subjects in emergency situations namely, individuals unable to provide prior informed consent or to receive preliminary trial information—remained limited (n = 133/7,449; 1.78%). More than half of these trials (n = 74/133; 55.64%) were sponsored by non-commercial entities, which showed increasing interest over time in research related to emergency care. This trend was particularly evident during the pandemic period, when a temporary increase in such studies was observed. Specifically, over the two-year period (2020–2021), a total of n = 55 trials were conducted, of which 31 trials sponsored by non-commercial entities (31/55; 56.36%), where the remaining 24 (n = 24/55; 43.64%) were industry-sponsored. Following the entry into force of CTR, a progressive and significant reduction in emergency trials has been noted. In 2025, only five trials involving participants in emergency situations were submitted (Table 3).

#### 3.2 Therapeutic areas

A total of 7,422 clinical trials were retrieved, for 27 studies, the sponsor did not select a therapeutic area. A total of 51 therapeutic areas were investigated across the analysed sample (n = 7,422), resulting in a total of n = 7,729 therapeutic area entries. It is important to note that the total number of therapeutic area entries (n = 7,729) exceeds the number of studies (n = 7,422) because the CTA allows multiple selections. Consequently, a single study may contribute more than one record to the database when multiple therapeutic areas are indicated. The analysis revealed a marked concentration of research topics, indicating that clinical research conducted in Italy has predominantly focused on a limited number of fields. For consistency, the evaluation focused on the thirteen most frequently represented therapeutic areas, which together accounted for 89.74% of all classifications (n = 6,936/7,729), categories were derived from MeSH headings and include disease groups as well as related physiological, diagnostic, and healthcare domains (Table 4). This uneven distribution highlights a pronounced polarization of experimental activity toward specific disease areas, while less represented fields remained analytically marginal.

**TABLE 4:** Number (n) and percentage (%) of therapeutic areas studied in clinical trials (CTs), 2016–2025.

Therapeutic areas	Number (n)	n/N %
Diseases [C] - neoplasms [C04]	2,821	36.50%
Diseases [C] - nervous system diseases [C10]	673	8.71%
Diseases [C] - immune system diseases [C20]	524	6.78%
Diseases [C] - and lymphatic diseases [C15]	437	5.65%
Diseases [C] - cardiovascular diseases [C14]	425	5.50%
Diseases [C] - respiratory tract diseases [C08]	354	4.58%
Diseases [C] - digestive system diseases [C06]	328	4.24%
Diseases [C] - virus diseases [C02]	306	3.96%
Diseases [C] - skin and connective tissue diseases [C17]	236	3.05%
Diseases [C] - nutritional and metabolic diseases [C18]	234	3.03%
Diseases [C] - musculoskeletal diseases [C05]	222	2.87%
Diseases [C] - congenital, hereditary, and neonatal diseases and abnormalities [C16]	206	2.67%
Diseases [C] - eye diseases [C11]	170	2.20%
Diseases [C] - female urogenital diseases and pregnancy complications [C13]	136	1.76%
Diseases [C] - hormonal diseases [C19]	96	1.24%
Diseases [C] - bacterial infections and mycoses [C01]	87	1.13%
Psychiatry and psychology [F] - mental disorders [F03]	87	1.13%
Phenomena and processes [G] - immune system processes [G12]	53	0.69%
Diseases [C] - pathological conditions, signs and symptoms [C23]	38	0.49%
Phenomena and processes [G] - metabolism [G03]	31	0.40%
Phenomena and processes [G] - genetic phenomena [G05]	30	0.39%
Analytical, diagnostic and therapeutic techniques and equipment [E] - diagnosis [E01]	27	0.35%
Analytical, diagnostic, therapeutic techniques and equipment [E] - anesthesia and analgesia [E03]	24	0.31%
Analytical, diagnostic and therapeutic techniques and equipment [E] - therapeutics [E02]	22	0.28%
Phenomena and processes [G] - musculoskeletal and neural physiological phenomena [G11]	17	0.22%
Phenomena and processes [G] - immune system phenomena [G13]	14	0.18%
Phenomena and processes [G] - physiological processes [G07]	14	0.18%
Diseases [C] - otorhinolaryngologic diseases [C09]	12	0.16%
Phenomena and processes [G] - digestive system and oral physiological phenomena [G10]	11	0.14%
Analytical, diagnostic, therapeutic techniques equipment [E]-Surgical procedures, operative [E04]	11	0.14%
Phenomena and processes [G] - reproductive and urinary physiological phenomena [G08]	10	0.13%
Psychiatry and psychology [F] - behavior and behavior mechanisms [F01]	9	0.12%
Diseases [C] - disorders of environmental origin [C21]	7	0.09%
Psychiatry and psychology [F] - behavioral disciplines and activities [F04]	6	0.08%
Analytical, diagnostic and therapeutic techniques and equipment [E] - investigative techniques [E05]	6	0.08%
Phenomena and processes [G] - circulatory and respiratory physiological phenomena [G09]	5	0.06%
Phenomena and processes [G] - ocular physiological phenomena [G14]	5	0.06%
Healthcare [N] - healthcare quality, access, and evaluation [N05]	4	0.05%
Diseases [C] - stomatognathic diseases [C07]	4	0.05%
Phenomena and processes [G] - physical phenomena [G01]	4	0.05%
Phenomena and processes [G] - biological phenomena [G16]	4	0.05%
Healthcare [N] - environment and public health [N06]	4	0.05%
Healthcare [N] - population characteristics [N01]	3	0.04%
Phenomena and processes [G] - cell physiological phenomena [G04]	2	0.03%
Psychiatry and psychology [F] – Psychological phenomena [F02]	2	0.03%
Phenomena and processes [G] - mathematical concepts [G17]	2	0.03%
Phenomena and processes [G] - microbiological phenomena [G06]	2	0.03%
Analytical, diagnostic and therapeutic techniques and equipment [E] - dentistry [E06]	1	0.01%
Healthcare [N] - healthcare economics and organizations [N03]	1	0.01%
Phenomena and processes [G] - chemical phenomena [G02]	1	0.01%
Analytical, diagnostic and therapeutic techniques and equipment [E] - equipment and supplies [E07]	1	0.01%
N Total	7,729	100.00%

Among the thirteen most represented therapeutic areas (n = 6,936 entries), oncology emerged as the leading research domain (n = 2,821/6,936; 40.67%). While maintaining the highest volume, the trend fluctuated significantly and showed a gradual decline in its relative share: from 39.59% in 2016 (n = 253/639) to a peak observed in 2021 (n = 353/794; 44.46%) followed by a post-2021 reduction and a subsequent recovery in 2025 (n = 324/834; 38.85%). Collectively, these peaks accounted for 24.00% of the entire category (n = 677/2,821).

The second most frequently investigated area was nervous system disorders (n = 673/6,936; 9.70%), which displayed moderately high values, reaching a peak in 2022 (n = 83/686; 12.10%). The trend was irregular, with marked fluctuations but an overall decreasing trajectory. Research on immune system diseases (n = 524/6,936; 7.55%), after an initial phase of limited activity, showed a sustained increase starting in 2021 (n = 62/794; 7.81%), culminating in a peak in 2025 (n = 82/834; 9.83%). Hemic and Lymphatic diseases (n = 437/6,936; 6.30%) ranked fourth in frequency and, after peaking in 2016 (n = 50/639; 7.82%) and 2021 (n = 55/794; 6.93%), exhibited a consistent decline in subsequent years. Cardiovascular research (n = 425/6,936; 6.13%) demonstrated a continuous upward trend, with a maximum observed in 2025 (n = 67/834; 8.03%). The respiratory disease area (n = 354/6,936; 5.10%) was characterised by marked variability, with a significant increase during the 2020–2024 period, maintaining a sustained level of post-pandemic relevance. This followed a peak in 2020 (n = 55/730; 7.53%), coinciding with the COVID-19 pandemic, and a second peak in 2024 (n = 61/747; 8.17%). In contrast, viral diseases (n = 306/6,936; 4.41%), after an isolated peak in 2020 (n = 88/730; 12.05%), showed a sharp normalization in the following years dropping to 2.64% by 2025 (n = 22/834). Digestive System diseases (n = 328/6,936; 4.73%), after peaking in 2024 (n = 48/747; 6.43%), normalized in 2025 (n = 33/834; 3.96%). Research in the Skin and Connective Tissue diseases area (n = 236/6,936; 3.40%) had low-to-medium volumes but showed growth; it demonstrated a fluctuating pattern up to 2021 (n = 18/794; 2.27%), after which a progressive increase was observed, reaching a maximum in 2024 (n = 38/747; 5.09%). A similar trend was noted for Nutritional and Metabolic diseases (n = 234/6,936; 3.37%), with a positive trajectory beginning in 2023 (n = 28/672; 4.17%) and being confirmed again in 2025 (n = 33/834; 3.96%). Musculoskeletal diseases (n = 222/6,936; 3.20%) had two peaks, in 2016 (n = 30/639; 4.69%) and in 2025 (n = 30/834; 3.60%), with a minimum reached in 2020 (n = 15/730; 2.05%). Areas that remained substantially stable without structural variations included Congenital, Hereditary, and Neonatal diseases (n = 206/6,936; 2.97%), with a peak in 2025 (n = 31/834; 3.72%), and Eye diseases (n = 170/6,936; 2.45%), which peaked in 2023 (n = 22/672; 3.27%).

**FIGURE 5:**
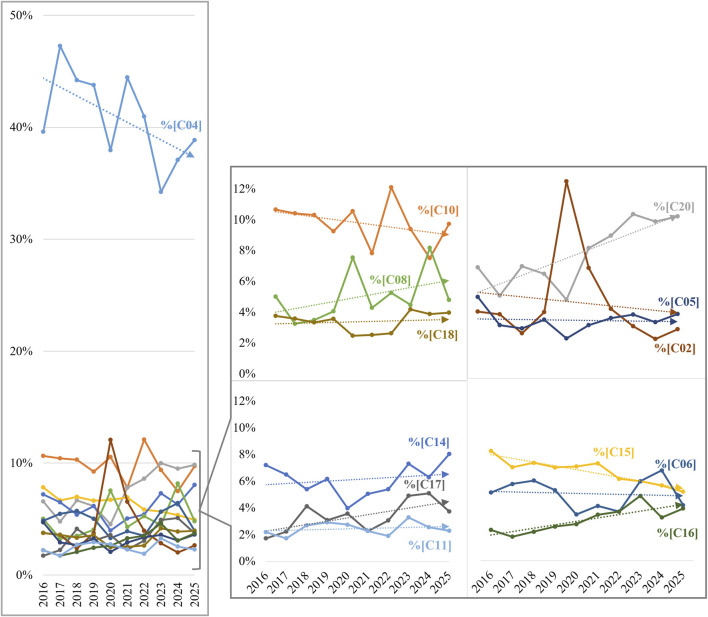
Percentage of most frequent therapeutic areas (% TAs) per year and trends (dotted line), 2016–2015. Abbreviations: C02, Virus Diseases; C04, Neoplasms; C05, Musculoskeletal Diseases; C06, Digestive System Diseases; C08, Respiratory Tract Diseases; C10, Nervous System Diseases; C11, Eye Diseases; C14, Cardiovascular Diseases; C15, Hemic and Lymphatic Diseases; C16, Congenital, Hereditary, and Neonatal Diseases and Abnormalities; C17, Skin and Connective Tissue Diseases; C18, Nutritional and Metabolic Diseases; C20, Immune System Diseases.

The annual percentage distribution of therapeutic areas, normalized to the total number of areas per year, between 2016 and 2025 reveals distinct and heterogeneous growth trajectories, highlighting shifting priorities within the biomedical and clinical research landscape, which is clearly illustrated in Figure 5.

#### 3.2.1 Trend in therapeutic areas

##### 3.2.1.1 High-grow therapeutic areas

Immune System diseases exhibited the most pronounced increase over the study period, rising sharply from relatively moderate baseline levels to become the fastest-growing therapeutic area by 2025. This steep upward trend suggests a substantial expansion of research and development activities, likely reflecting advances in immunotherapies, biologics, and immune-mediated disease management.

A strong and consistent increase was also observed for cardiovascular diseases, which demonstrated a stable linear growth throughout the entire period.

Respiratory Tract diseases showed a marked upward trajectory, with growth accelerating particularly after 2019. The level of ongoing activity showed continued research in this space which peaked in 2024.

##### 3.2.1.2 Moderate but consistent growth

Several therapeutic areas displayed steady, moderate increases over time. Congenital, Hereditary, and Neonatal diseases showed continuous growth, reflecting expanding interest in genetic disorders, rare diseases, and precision medicine approaches.

Similarly, Digestive System diseases and Skin and Connective Tissue diseases demonstrated progressive upward trends, indicating gradual but sustained expansion in these domains. Musculoskeletal diseases and Eye diseases also exhibited linear growth, albeit with lower slopes, suggesting stable research activity without abrupt shifts.

##### 3.2.1.3 Areas with stability or decline

In contrast, Neoplasms, while it remains the most researched field by volume, saw its relative percentage share has followed a downward slope over the decade.

Hemic and lymphatic diseases showed a gradual decline over the observed period, indicating a relative reduction in activity or a redistribution of focus toward other therapeutic areas. A comparable downward trend was evident for Virus diseases, which after the peak in 2020–2022 returned to pre-pandemic activity.

##### 3.2.1.4 Low but positive growth areas

Nutritional and Metabolic diseases displayed modest yet consistent growth, maintaining relatively low absolute values but showing a positive linear trend. This pattern suggests steady interest without major expansion.

#### 3.2.2 Phase

**FIGURE 6:**
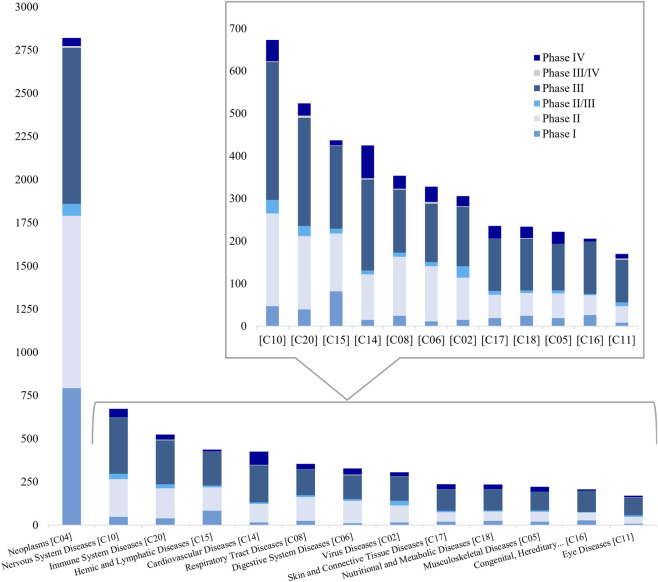
Most frequent therapeutic areas (TAs) by phase. The zoomed-in section clarifies the phase distribution for most frequent TAs other than neoplasm.

Phase III trials predominated across all therapeutic areas except oncology, where Phase II (n = 998/2,821; 35.38%) and Phase I (n = 793/2,821; 28.11%) studies were most frequent. Hemic and Lymphatic diseases, followed by the Congenital, Hereditary, and Neonatal diseases and Abnormalities category, represented the most common areas for Phase I trials after oncology (n = 82/437; 18.76% and n = 26/206; 12.62%). It is interesting to note that Phase IV studies are concentrated in cardiovascular diseases (n = 77/425; 18.12%), followed by skin diseases (n = 30/236; 12.71%), and musculoskeletal disorders (n = 28/222; 12.61%) (Figure 6).

#### 3.2.3 Sponsor profile

Commercial sponsorship was predominant overall, accounting for 78.20% of therapeutic areas (n = 5,424/6,936), while non-commercial initiatives represented only 21.80% (n = 1,512/6,936).

The degree of academic involvement, however, varied by therapeutic area. In oncology, the proportion of non-commercial studies remained marginal (n = 672/2,821; 23.82%), mirroring trends observed across most other disease areas. Greater academic participation was recorded in cardiovascular diseases (n = 161/425; 37.88%), viral diseases (n = 113/306; 36.93%), and hematologic/lymphatic disorders (n = 109/437; 24.94%).

The reliance on commercial entities was particularly pronounced in several disease categories, including Nervous System Diseases (n = 570/673; 84.70%), Immune System Diseases (n = 469/524; 89.50%), Respiratory Tract Diseases (n = 272/354; 76.84%), and Digestive System Diseases (n = 268/328; 81.71%). In contrast, non-commercial activity was notably low in Skin and Connective Tissue Diseases, accounting for only 11.86% (n = 28/236) of studies. Moderate levels of non-commercial involvement were observed in Nutritional and Metabolic Diseases (n = 51/234; 21.79%) and Musculoskeletal Diseases (n = 51/222, 22.97%).

**FIGURE 7:**
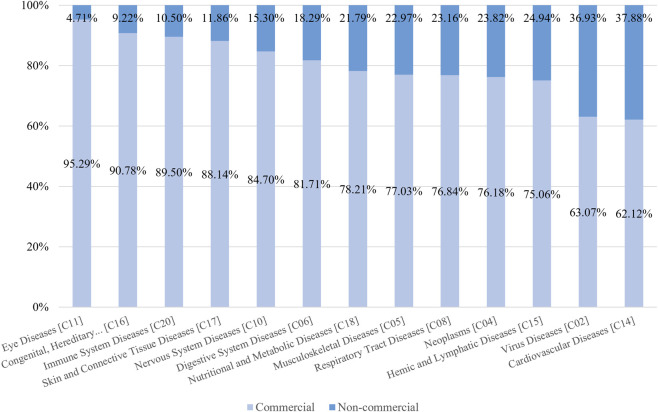
Proportional distribution of most frequent therapeutic areas (TAs) by sponsor profile (commercial vs. non-commercial) presented as a 100% stacked bar chart, 2016–2015.

In contrast, the lowest levels of non-commercial activity were observed in Congenital, Hereditary, and Neonatal diseases and Abnormalities (n = 19/206; 9.22%), and finally Eye diseases (n = 8/170; 4.71%) (Figure 7).

#### 3.2.4 Study population

##### 3.2.4.1 Age

**FIGURE 8:**
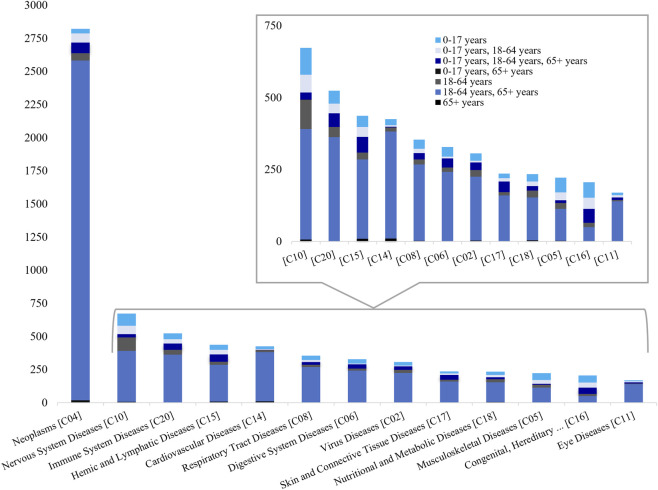
Most frequent therapeutic areas (TAs) by age group. The zoomed-in section clarifies the distribution for most frequent TAs other than neoplasm.

The analysis reveals a distribution of therapeutic areas heavily concentrated in the adult and elderly population (18–64 years, 65+ years) (n = 5,298/6,936; 76.38%), but with some very marked therapeutic specializations for younger age groups (Figure 8). Specific dynamics emerge for various pathologies:

Oncology: This area was almost exclusively focused on adults and the elderly (n = 2,565/2,821; 90.93%), while recording only minimal inclusion of exclusively paediatric populations (n = 34/2,821; 1.21%).

Paediatric research: Therapeutic areas studied exclusively involving paediatric populations (0–17 years) were rare (n = 477/6,936; 6.88%), as they are typically embedded within multi-age cohorts (n = 1,221/6,936; 17.60%). These figures reflect the number of therapeutic area classifications, not the number of individual clinical trials; the corresponding count of CTAs enrolling exclusively paediatric participants is reported in Section 3.1.3 (n = 565/7,442; 7.59%)

Following the Congenital, Hereditary, and Neonatal diseases and Abnormalities area (n = 54/206; 26.21%)—which represents the category with the highest “youth density”—the areas where minors have the greatest weight relative to the category’s total volume were Musculoskeletal diseases (n = 51/222; 22.97%), Nervous System diseases (n = 93/673; 13.82%), and Digestive System diseases (n = 33/328; 10.06%).

The most unusual finding is the near-absence of therapeutic areas studied exclusively in the 65+ years group (n = 56/6,936; 0.81%), especially in areas such as cardiovascular diseases (n = 10/425; 2.35%) or respiratory diseases (n = 2/354; 0.56%). Furthermore, this is the only age group in which a clear predominance of commercial sponsorship is not observed. In this segment, for six therapeutic areas, research activity appears to be predominantly or significantly supported by academic sponsors or non-commercial entities.

##### 3.2.4.2 Sex

All therapeutic areas were studied in both sexes (n = 6,432/6,936; 92.73%), while male-only (n = 247/6,936; 3.56%) or female-only (n = 257/6,936; 3.71%) trials were rare.

Specific patterns emerged within certain domains:

Oncology: While the vast majority included both sexes (n = 2,482/2,821; 87.98%), 7.48% (n = 211/2,821) of studies focused exclusively on females, whereas male-only trials accounted for 4.54% (n = 128/2,821).

Hemic and Lymphatic diseases and Digestive System diseases: No female-only studies were identified. Notably, Hemic and Lymphatic diseases showed 36 male-only trials (n = 36/437; 8.24%), while Digestive System diseases had only 2 (n = 2/328; 0.61%).

Respiratory Tract diseases, Skin and Connective Tissue diseases, and Eye diseases: No male-only studies were found. The volume of female-only studies remained low across these areas, with two trials in Respiratory (n = 2/354; 0.56%), 6 in Skin (n = 6/236; 2.54%), and only one in Eye diseases (n = 1/170; 0.59%).

Musculoskeletal diseases: This area presented a curious male prevalence in single-sex studies, with 39 “male-only” trials (n = 39/222; 17.57%) and only 3 “female-only” trials (n = 3/222; 1.35%), despite being a field—much like osteoporosis—often associated with the female sex. Other Notable Trends: Nervous System and Immune System Diseases also showed a high reliance on mixed-sex cohorts (n = 653/673; 97.03% and n = 518/524; 98.85% respectively). Congenital and Hereditary Diseases showed a relatively higher proportion of male-only trials (n = 23/206; 11.17%) compared to female-only trials (n = 1/206; 0.49%).

##### 3.2.4.3 Vulnerable populations

The 13 most represented therapeutic areas are only limitedly studied in pregnant or breastfeeding women (n = 68/6,936; 0.98%). The majority involve therapeutic areas studied in both pregnant and breastfeeding women (n = 28), with 6 recorded in 2020, or exclusively pregnant women (n = 29).

Viral diseases are the predominant area (n = 21/68; 30.88%), followed by Respiratory diseases (n = 10) and Neoplasms (n = 9). These study areas reflect pathologies with the highest risk of transmission or impact during gestation: specific research exclusively on breastfeeding women is the rarest, with only 11 total cases across all therapeutic areas.

Research on incapacitated subjects (n = 276/6,936; 3.98%) is most active in neoplasms (n = 95/276; 34.42%), followed by Nervous System diseases (n = 62/276; 22.46%). The latter is an expected category, as it includes pathologies such as Alzheimer’s, advanced Parkinson’s, or traumatic brain injuries.

Additionally, Respiratory Tract diseases (n = 30/276; 10.87%) complete the top three areas, which together cover 67.75% of the total research involving this vulnerable population.

Other notable areas include Virus Diseases (n = 26/276; 9.42%), Cardiovascular Diseases (n = 24/276; 8.70%), while categories like Digestive System Diseases (n = 8/276; 2.90%) and Immune System Diseases (n = 11/276; 3.99%) show lower research volumes.

A total of 110 (n = 110/6,936; 1.59%) cases involved subjects in emergency situations. Emergency-related research focused predominantly on two areas: Respiratory Tract and Cardiovascular Diseases (n = 25/110; 22.73% each). These findings reflect the clinical priorities within Emergency Department and Intensive Care Unit, such as acute respiratory failure, myocardial infarctions, fulminant viral infections, and neurological trauma.

These were followed by Virus Diseases (n = 19/110; 17.27%) and Nervous System Diseases (n = 10/110; 9.09%) and Neoplasms (n = 9/110; 8.18%). Furthermore, Hemic and Lymphatic Diseases represented 7.27% (n = 8/110) of the emergency cases. In contrast, areas such as Musculoskeletal or Eye Diseases showed zero activity in emergency research contexts.

#### 3.3 Investigational medicinal product

In the period between 2016 and 2025, a total of 22,991 IMPs were tested across the 7,449 studies submitted in Italy. These can be grouped into four categories: Chemical, Biological, ATMPs and ADCs, which are composed of an antibody linked to a cytotoxic “payload.” It is important to emphasize that these values refer to test IMPs and comparator IMPs (auxiliary IMPs were excluded) and not to the number of CTAs.

For this specific analysis, the data also included CTAs related to IMPD-Q (Quality data), bringing the total number of CTAs considered for IMP analysis to 7,581. Furthermore, a single study may contribute multiple entries to the database—for instance, in the presence of different formulations or dosages.

The longitudinal analysis of IMPs from 2016 to 2025 reveals a steady shift in the pharmaceutical landscape, characterised by the progressive growth of biological entities and fluctuations in sponsorship distribution.

The market reached a peak in activity in 2021 (n = 3,096/22,991; 13.47%), followed by a contraction in the 2023–2024 biennium (n = 1,753/22,991; 7.62% and n = 1,823/22,991; 7.93%, respectively) and a recovery in 2025 (n = 2,243/22,991; 9.76%).

Chemical IMPsThese represented the prevailing component (n = 15,579/22,991; 67.76%). While 2021 recorded the highest absolute value (n = 2,083/3,096; 67.28%) and 2019 the highest percentage (n = 1,669/2,315; 72.10%), 2024 marked the lowest point of utilization (n = 1,166/1,823; 63.96%). Although a slight recovery was observed in 2025 (n = 1,401/2,243; 62.46%), the overall trend decreased, failing to return to pre-2021 levels. Overall, chemical products dropped from 71.34% (n = 1,516/2,125) in 2016 to 62.46% (n = 1,401/2,243) in 2025.

**FIGURE 9:**
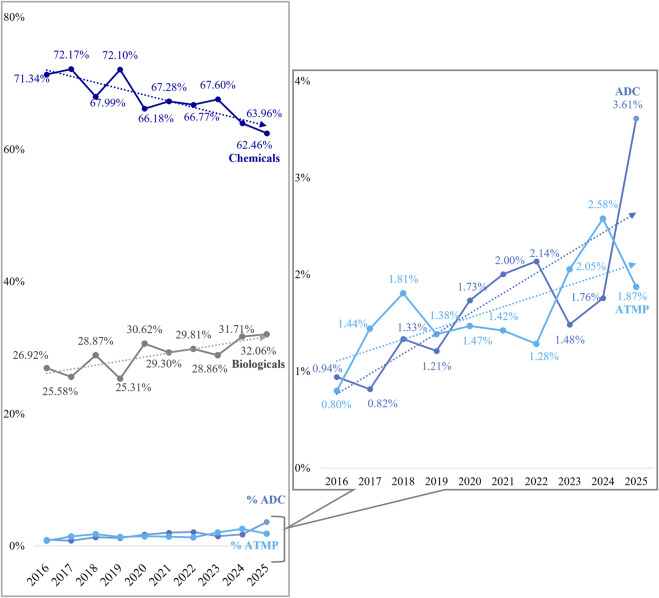
Percentage of IMP category (% IMPs) per year and trends (dotted lines), 2016–2025. The inset (right) provides a detailed view for ATMP and ADC.

Biological IMPsThis was the second most represented category (n = 6,652/22,991; 28.93%). They showed a significant increase during the pandemic period, and even the contraction in 2023 (n = 506/1,753; 28.86%) did not halt their growth. Over the decade, they rose from 26.92% (n = 572/2,125) in 2016 to a historical peak of 32.06% (n = 719/2,243) in 2025 (Figure 9).

ADCs, although remaining marginal overall (n = 398/22,991; 1.73%), have more than tripled in frequency, rising from a historical average of 1%–3.61% in 2025. This was marked by a record surge of 81 IMPs submitted in the final year (n = 81/2,243; 3.61%).

ATMPs, while numerically fewer (n = 362/22,991; 1.57%), have also doubled their presence, growing from 0.80% in 2016 (n = 17/2,125) to 1.87% in 2025 (n = 42/2,243), having reached a peak of activity in 2024 (n = 47/1,823; 2.58%) (Figure 9).

The market is evolving from the near-exclusivity of chemical agents toward a more balanced model, where biological therapies and chemical-biological combinations gain ground year after year.

##### 3.3.1 Phase

**FIGURE 10:**
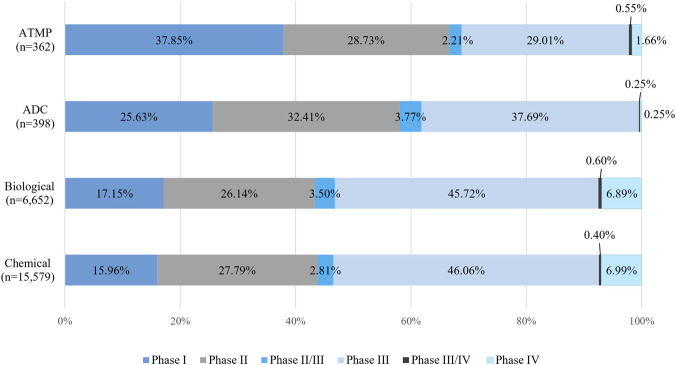
Total count (n) of IMP categories by phase presented as a 100% stacked bar chart, 2016–2015.

While Chemical and Biological IMPs display a “complete” structure (ranging from Phase I to IV), ATMPs and ADCs are clearly oriented toward the future, with very few products in post-marketing stages (Phase IV) (Figure 10).

The Chemical IMPs held the highest volumes in absolute terms and showed a strong concentration in advanced phases. Phase III represented the largest segment for this category (n = 7,176/15,579; 46.06%). It also features the highest number of products in Phase IV (n = 1,089/15,579; 6.99%) compared to all other categories, indicating a mature and consolidated market.

The distribution of Biological IMPs followed a trend similar to that of chemicals, with a prevalence of Phase III (n = 3,041/6,652; 45.72%). However, compared to chemicals, they have a significant proportion in Phase I (n = 1,141/6,652; 17.15%) and Phase II (n = 1,739/6,652; 26.14%).

In contrast, ATMPs and ADCs, despite having lower total volumes, showed interesting percentage distributions.

ATMPs: This category is heavily skewed toward early phases. Phase I (n = 137/362; 37.85%) is preponderant, signaling an emerging sector with many products still in early experimental stages.

ADCs: These presented a more balanced distribution between Phase I (n = 102/398; 25.63%), Phase II (n = 129/398; 32.41%), and Phase III (n = 150/398; 37.69%), with an almost zero presence in Phase IV (n = 1/398; 0.25%)—a sign of an innovative technology that is only now reaching commercial maturity.

“Hybrid” phases (Phase II/III and Phase III/IV) are numerically the least frequent across all IMP types.

##### 3.3.2 Sponsor profile

The analysis of IMP distribution relative to sponsor profile (commercial vs. non-commercial) highlights a clear prevalence of industrial investment across all technological categories.

**FIGURE 11:**
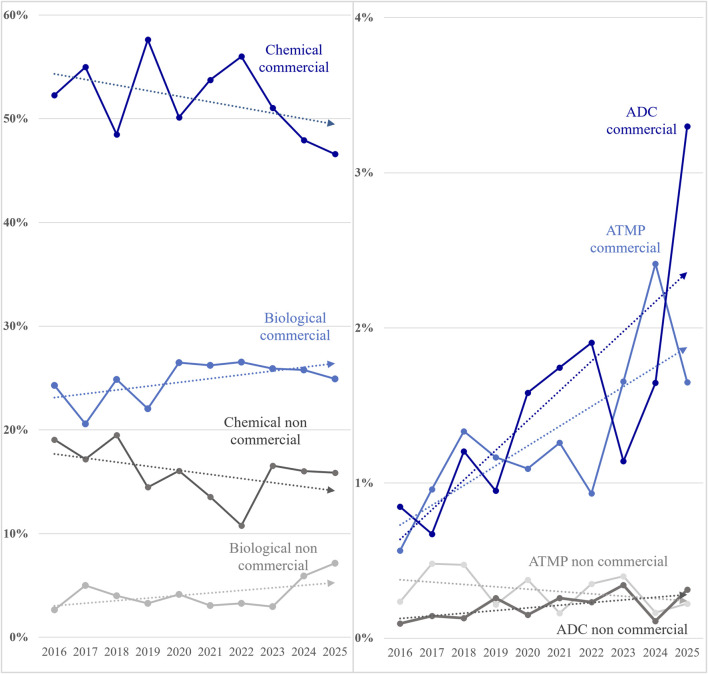
Percentage of IMP category (% IMPs) per year and trends (dotted lines), by sponsorship type (commercial or non-commercial), 2016–2025. The left panel shows established modalities (Chemical and Biological), while the right panel provides a magnified scale view of emerging modalities (ADC and ATMP).

Out of 22,991 IMPs identified and categorised, the majority were associated with commercial sponsors (n = 18,325/22,991; 79.71%), while non-commercial entities accounted for 20.29% (n = 4,666/22,991) of the total (Figure 11).

Commercial chemical IMPs constituted the largest single segment, accounting for 52.06% of the total dataset (n = 11,968/22,991). Within the chemical category, 76.82% of IMPs were supported by commercial sponsors (n = 11,968/15,579), while this group also showed the highest share of non-commercial sponsorship (n = 3,611/15,579; 23.18%). This pattern is likely due to lower production costs and the wide availability of known molecules used in independent academic research.

Commercial chemical sponsorship reached its peak in 2019 (n = 1,334/2,315; 57.62%) before declining to 46.59% in 2025 (n = 1,045/2,243).

Non-commercial chemical IMPs showed greater variability across years, ranging from 10.77% (n = 277/2,573) in 2022 to 19.49% in 2018 (n = 453/2,324), reflecting the continued relevance of academic and investigator-driven trials using established or repurposed chemical compounds.

Conversely, biological IMPs (n = 5,714/6,652; 85.90%) and ADCs (n = 351/398; 88.19%) demonstrated a markedly higher prevalence of industrial sponsorship.

A significant peak was observed in the non-commercial biological sector, which reached its highest point in 2025 (n = 160/2,243; 7.13%), more than doubling its share compared to 2016 (n = 56/2,125; 2.64%).

Although ATMPs constitute a smaller fraction of the total IMPs, they still exhibited a strong commercial orientation (n = 292/362; 80.66%). Nevertheless, non-commercial involvement remained notable (n = 70/362; 19.34%) reflecting a relatively greater contribution of academic or non-commercial research in this highly innovative field. These datasuggest that universities and clinical centers of excellence still play a fundamental role in the early development stages of cell and gene therapies (Figure 11).

##### 3.3.3 Study population

###### 3.3.3.1 Age

The cross-analysis between IMP types and the age of the study population reveals a clear prevalence of studies involving adult and elderly participants (18–64 years and ≥65 years).

Adult and elderly populations (18–64 years, 65+ years) dominated all categories, particularly for chemical drugs (n = 12,300/15,573; 78.98%) and biological agents (n = 4,941/6,650; 74.30%). This age group also represented the vast majority of ADCs (n = 375/398; 94.22%) and more than half of ATMPs (n = 205/362; 56.63%)

Regarding paediatric involvement (0–17 years), the biological sector showed a proportionally higher presence in the paediatric-only cohort (n = 705/6,650; 10.60%) relative to its total volume, suggesting a strong application of biotechnology in paediatrics. In contrast, ATMPs and Chemical drugs showed lower paediatric-only participation, accounting for 8.84% (n = 32/362) and 5.82% (n = 906/15,573), respectively.

A notable portion of ATMP studies (n = 45/362; 12.43%) and biological trials (n = 396/6,650; 5.95%) included participants across the entire age spectrum (0–17, 18–64, and 65+ years), indicating broader age inclusivity within these categories.

Chemical IMPs were the only category evaluated in an intergenerational population (0–17 and 65 years), with only a single occurrence recorded over the entire observation period.

Trials involving exclusively elderly participants (65+ years) were rare across all IMP categories. Chemical drugs accounting for the highest absolute number (n = 173/15,573; 1.11%), while ATMPs had no recorded trials in elderly populations.

###### 3.3.3.2 Sex

For this analysis, seven clinical trials were excluded due to a lack of information regarding the sex of the population.

It is interesting to note that among the IMPs tested on a single sex, those dedicated exclusively to women (n = 1,151/22,984; 5.01%) were nearly double those dedicated exclusively to men (n = 682/22,984; 2.97%).

Within the ATMP category, mixed-sex cohorts represented the vast majority (n = 327/362; 90.33%), while trials dedicated exclusively to men (n = 29/362; 8.01%) were more frequent than those for women (n = 6/362; 1.66%).

In the Biological IMP and ADC categories, although mixed-sex cohorts dominated (n = 6,286/6,650; 94.53% and n = 360/398; 90.45%, respectively), there was a marked disproportion toward the female gender (n = 218/6,650; 3.28% and n = 36/398; 9.05%, respectively) compared to the male gender (n = 146/6,650; 2.20% and n = 2/398; 0.50%, respectively).

Finally, for Chemical IMPs, most trials involved both sexes (n = 14,178/15,574; 91.04%), but those focused exclusively on women (n = 891/15,574; 5.72%) significantly outnumbered those focused exclusively on men (n = 505/15,574; 3.24%).

###### 3.3.3.3 Vulnerable populations

The participation of pregnant or breastfeeding women (n = 209/22,991; 0.91%) varies significantly based on the technological nature of the drug.

Chemical IMPs: This is the most studied category in women who are exclusively pregnant (n = 63/209; 30.14%) or pregnant in association with nursing (n = 41/209; 19.62%). This reflects the deeper historical data and the broad spectrum of applications of traditional chemical compounds.

Biological Drugs: These show a balanced presence for pregnant women (n = 29/86; 13.88%) and breastfeeding women (n = 16/86; 7.66%). Notably, this is the only category to have a higher number of studies specifically on breastfeeding compared to chemical drugs (n = 15/209; 7.18%).

ATMPs: Involvement is nearly non-existent, with only 4 IMPs studied in pregnancy (n = 1) or across both categories (n = 3). This is consistent with the experimental and complex nature of these therapies, which are often initially tested on less vulnerable populations.

ADCs: This category was never studied in these specific populations over the decade, confirming a research profile that remains highly selective.

###### 3.3.3.4 Emergency situations

IMPs studied in emergency contexts represent a marginal and rare share (n = 299/22,991; 1.30%).

The internal percentage distribution is as follows:

Chemical IMPs: 73.6% (n = 220/299).

Biological IMPs: 20.7% (n = 62/299).

ATMPs: 5.3% (n = 16/299).

ADCs: A single occurrence in this specific context (n = 1/299; 0.3%).

Although Chemical IMPs present the highest absolute number of emergency events, this result is influenced by their substantially larger presence in the overall dataset (n = 15,579). When the relative incidence is considered, a different pattern emerges. ATMPs show the highest proportional occurrence of emergency situations (n = 16/362; 4.42%), which is markedly higher than the relative incidence observed for Chemical IMPs (n = 220/15,579; 1.41%), Biological IMPs (n = 62/6,652; 0.93%), and ADCs (n = 1/398; 0.25%).

## 4 Discussion

The analysis of clinical trial submissions to AIFA during the 2016–2025 period presents the picture of an overall resilient system undergoing a profound structural transformation. The apparent stability of total volumes—averaging approximately 745 trials per year—masks divergent dynamics between sponsor profiles, therapeutic areas, study populations, and experimental technologies that are substantially redefining the national clinical research landscape.

The Italian system has demonstrated a remarkable capacity to absorb European CTR and national regulatory changes. After the exceptional peak in 2021 linked to the pandemic, the decline observed in the 2022–2023 biennium (688 trials in 2023) coincided with the implementation of the new Ethics Committee structure and the full adoption of the CTIS portal. In Italy, 2023 was the year of the enactment and implementation of new national regulations aimed at reorganizing the Ethics Committee system and ensuring administrative alignment.

The recovery recorded in 2025, with 838 clinical trials (the second-best result of the decade), suggests that the system has moved past the learning curve and entered a phase of greater maturity. In this context, Italy stands out as one of the most active European countries for clinical trial activity within the EEA (IQVIA-EFPIA-VE, 2024), contributing significantly to the continent’s critical mass despite the relative decline in Western Europe’s attractiveness (IQVIA Institute for Human Data Science, 2025; European Union, 2018; Questions and answers on the european biotech act, 2026).

### 4.1 Quantitative stability and structural reconfiguration

The maintenance of overall volumes, however, hides a significant structural shift: the growing divergence between commercial and non-commercial research. During the observed period, the industrial sector showed marked resilience, recovering quickly in the post-pandemic era and exceeding pre-COVID levels by 2025.

The predominance of Phase II and III trials confirms that Italy is primarily attractive for pivotal and confirmatory studies, in line with the strength of its clinical network and the high quality of its investigational sites. However, the steady growth of Phase I trials, although still limited in absolute numbers, represents a positive and relatively distinctive signal, indicating a progressive strengthening of early-stage and first-in-human research infrastructures. By contrast, the structural decline of Phase IV studies—particularly within the non-commercial segment—suggests reduced attention to independent post-marketing research, which is a critical issue for the Italian public healthcare system.

Conversely, non-commercial research highlights a progressive and structural decline: from 204 studies in 2016 to 158 studies in 2025, with a historical low reached in 2023 (130 studies). In relative terms, the share of independent research in Italy fell from 28.18% (n = 204/724) in 2016 to 18.85% (n = 158/838) in 2025, signalling a systemic loss of influence.

This dynamic may suggests that the CTR, while introducing a harmonized regulatory framework, has had an asymmetric impact on system stakeholders. Industry has been able to capitalize on the centralization of procedures and regulatory predictability, whereas universities, public hospitals, and non-commercial entities appear less equipped to sustain the increase in administrative burdens, costs, and management complexity.

In this context, the Ministerial Decree of 30 November 2021 (which entered into force in March 2022) was designed to support non-commercial research and encourage the utilization of data for regulatory purposes, including through collaborations with industry (Italian Ministry of Health, 2022). However, temporal analysis shows that the Decree may have not produced an immediate or sufficient impact on the non-commercial sector’s operational capacity, likely due to administrative complexities, infrastructural deficiencies, and delays in adopting new mechanisms. The Decree alone has not closed the growing gap with industry.

EMA reports indicate that approximately 40% of European submissions in 2025 concern mono-national studies, which are often non-commercial (Monitoring the European clinical trials environment, 2025a; Monitoring the European clinical trials environment, 2025b; Monitoring the European clinical trials environment, 2025c; Monitoring the European clinical trials environment, 2025d). These data highlight that the crisis of independent research is a relevant issue at the European level however, in Italy, the phenomenon appears more pronounced than the average of the main European indicators. The Italian academic system struggles to transform independent research into advanced clinical development initiatives or biotech start-ups, partly due to bureaucratic constraints and the high cost of drugs and complex technologies.

### 4.2 Technological transition and representative criticalities

The evolution of the IMP portfolio highlights a clear biotechnological transition. Although small molecules remain prevalent in absolute terms, their relative share is in constant decline, in line with the global trend, which highlights a decrease in small molecules in advanced-phase trials. Meanwhile, biologicals reached a historical peak of 32.06% in 2025, exceeding the linear growth predicted internationally (IQVIA Institute for Human Data Science, 2025).

The emergence of ADCs and specialization in advanced therapies is particularly relevant. The most innovative phenomenon of the 2023–2025 triennium is represented by the rise of ADCs, particularly in oncology. Globally, as of 2025, there are 21 approved ADCs and over 500 ongoing trials (Wang et al., 2025; Wu et al., 2025), with a significant impact on the prognosis of high-prevalence neoplasms such as breast cancer. In Italy, ADCs represent 3.61% of IMPs in 2025 with 81 active molecules, a share that places the country among the early adopters of this technology compared to the world average. These data suggest an advanced specialization of Italian sites in managing drugs with high biological selectivity and production complexity.

The European Commission’s Biotech Act (December 2025) (European Union, 2018), aimed at reducing the time-to-market for biotechnologies and bridging the global competitive gap, Italian data suggest that the national clinical infrastructure is ready to embrace this change of speed. However, alongside these elements of excellence, significant criticalities emerge regarding representativeness: biologicals, ATMPs, and ADCs are almost exclusively tested on non-vulnerable adult populations, while pregnant/breastfeeding women and the “pure” elderly remain systematically underrepresented.

Chemical drugs are the only ones to show, albeit marginally, the involvement of pregnant and breastfeeding women, likely due to a greater understanding of their mechanisms of action and a historically better-defined risk profile. Conversely, biologicals, ATMPs, and ADCs are almost exclusively tested on non-vulnerable adult populations. This particularly cautious approach, induced by high biological and regulatory complexity, risks generating future gaps in clinical evidence precisely in the areas of greatest scientific advancement.

### 4.3 Vulnerable populations and gender medicine

Vulnerable populations remain underrepresented. Although a significant portion of clinical studies involves populations considered vulnerable in a regulatory sense (minors or incapacitated subjects), pregnant and breastfeeding women emerge as the least represented group overall. These data highlight a persistent perception of pregnancy as a “high-risk” condition, more difficult to integrate into research protocols than other forms of vulnerability that have now been “normalized.”

On the regulatory front, Italy possesses a formally advanced framework. Legislative Decree 52/2019 (Italian Republic, 2019), implementing Law 3/2018 (Italian Republic, 2018), introduced the obligation to consider gender differences in the design and conduct of clinical trials, transforming gender medicine from an ethical principle into a regulatory requirement. This is supported by the Istituto Superiore di Sanità (ISS) Plan for the Application and Diffusion of Gender Medicine (Gender medicine in Italy, 2016), which aims to promote fairer and more transparent research, with the goal of improving the Gender equality index (2025) and ensuring greater readability of clinical data from a gender perspective by 2026.

In particular, the inclusion of pregnant and breastfeeding women remains episodic and reactive, as demonstrated by the anomalous peak in 2020—the year the pandemic emergency made the need for rapid clinical evidence in these population groups imperative. The peak observed in 2020 represents a key element of interpretation: it demonstrates that in the presence of high and widespread clinical risk, the system can overcome ethical, regulatory, and organizational barriers normally considered insurmountable. However, the rapid return to minimal levels in subsequent years indicates that this opening did not translate into a structural change. Inclusion remains episodic, reactive, and context-dependent rather than guided by a long-term scientific strategy.

The regulatory transition currently underway, culminating in the publication of the ICH E21 draft guideline in June 2025 (European Medicines Agency, 2025), marks a fundamental paradigm shift, moving from a logic of preventive exclusion to protection through active evidence production. This new approach no longer mandates the automatic exclusion of participants who become pregnant during a study but promotes their retention in the trial through dedicated monitoring and risk management protocols. The stated objective is the production of solid and systematic data necessary for evidence-based clinical decisions, with full implementation expected by 2026.

In this context, the European Commission is actively seeking to support a more inclusive and risk-proportionate approach to clinical research, emphasizing the need to balance the risks of inclusion against those of exclusion, in line with the principles of the Declaration of Helsinki (WMA, 2024). By promoting regulatory flexibility, proportionate oversight, and the use of innovative trial designs and real-world data, the European Commission aims to facilitate the responsible inclusion of vulnerable populations, thereby improving the evidence base for populations that are routinely exposed to medicinal products in clinical practice (European Union, 2018).

### 4.4 Systemic implications and the role of independent research

Exclusively female trials represent 9.01% of non-commercial trials, a share nearly triple that observed in the commercial sector (3.36%). These data indicate a greater sensitivity of the academic world toward gender differences and the production of clinical evidence aimed at women’s health, in line with national regulatory principles on gender medicine.

A similar centrality emerges in geriatric-centric studies and congenital paediatric diseases—fields in which independent research acts as the main scientific engine.

A particularly relevant finding, and one that is partly paradoxical in the Italian context, is the extremely limited presence of studies exclusively dedicated to the population aged ≥65 years. Despite Italy being among the most long-lived countries worldwide, clinical research remains heavily focused on “standard” adult populations, with older adults almost invariably included within mixed-age cohorts and very few studies specifically designed to address geriatric needs. This imbalance emerges most prominently in the cardiovascular and respiratory fields, where the disease burden is highest in the elderly population. Notably, in this segment, the relative contribution of non-commercial research increases, suggesting an attempt by the academic community to fill a gap left by industry-sponsored research. While industry tends to favour standardized adult populations, the non-commercial sector maintains a significant commitment to studying the “pure” over-65s and congenital pathologies, areas of high clinical and organizational complexity but limited commercial attractiveness.

The growing concentration of clinical trials on industrial initiatives and advanced stages of development carries systemic risks. While it ensures rapid patient access to innovative therapies, it may reduce evidence production in areas less attractive to the market, such as non-patentable therapeutic strategies, ultra-rare conditions, or vulnerable populations. Historically, these areas have been protected by independent research, whose structural decline represents a key factor in perpetuating current knowledge gaps.

### 4.5 Future perspectives

Overall, the Italian system appears mature and competitive in Phases II–III, highly attractive to industry and capable of managing biotechnological innovation. However, weaknesses remain in the production of independent post-marketing evidence and in the inclusivity of study population. In an increasingly competitive global context, the success of the Italian system will depend on the ability to support scientific excellence with targeted operational and infrastructural interventions: bureaucratic simplification, structural strengthening of non-commercial research, and the full utilization of Real-World Evidence through digital tools such as the Electronic Health Record (Fascicolo Sanitario Elettronico).

Without a rebalancing among technological innovation, independent research and real clinical needs, there is a risk that scientific progress will proceed out of alignment with the principles of equity and sustainability that should guide the medicine of the future.

The structural decline of non-commercial research (which fell to 17.62% in 2022) requires urgent corrective interventions. It is desirable to implement virtuous mechanisms that allow the reinvestment into the National Health Service (SSN) of the economic benefits and savings generated by clinical trials, thereby guaranteeing academia the resources necessary to operate on Frontier technologies (e.g., ATMPs).

Digitalization and valorization of RWD: the widespread implementation of the Electronic Health Record represents the cornerstone for integrating traditional clinical research with real-world evidence. The systematic use of RWD would not only allow for better patient selection, reducing study failure rates, but would also position Italy as a leader in analyzing long-term health outcomes for new biotechnological therapies.

The review of the past decade demonstrates that historical analysis provides an essential basis for future policy and research planning. The technological transition toward biologicals is now a fact; the challenge for the Italian system now lies in the ability to evolve from a reactive to a proactive model, capable of attracting global investment through a modern digital infrastructure and streamlined governance, consistent with the ambitions of the European Biotech Act (European Union, 2018).

Hosting clinical trials confers multiple strategic advantages. A vibrant research and development ecosystem can improve population health, enhance the sustainability and resilience of healthcare systems, and drive economic development. Revitalising clinical research is strategically important not only for regulatory governance but also as an economic opportunity and a pathway to earlier access to innovative therapies. Reinforcing Europe’s clinical trial ecosystem could boost specialised employment, attract investment, and expand patient access to novel therapies, at a time when Europe’s share of multinational trials has declined in recent years.

## 5 Conclusion

The main findings highlight both areas of progress and persistent limitations. The Italian clinical trials landscape demonstrates strong resilience and sustained growth in the number of studies, with high appeal for industry-sponsored research, yet it remains structurally unbalanced.

The predominance of oncology and commercially sponsored trials reflects well-established priorities, while non-commercial activity across several therapeutic areas points to an underexploited potential. The growing role of biological products and ATMPs signals an evolution in scientific and clinical expertise, although challenges remain in sustaining these efforts and translating them into clinical practice. Notably, vulnerable populations such as pregnant and breastfeeding women remain systematically underrepresented. While a good balance and broad inclusion of both sexes was generally observed, sex-specific studies remained uncommon. Taken together, these patterns underscore the importance of continued methodological refinement and the adoption of more inclusive research strategies.

Future efforts should focus on strengthening infrastructure for non-commercial research, improving regulatory frameworks for the involvement of healthy volunteers and supporting continuity in the development of advanced therapies. Further monitoring will be essential to assess how recent policy and regulatory changes may shape the trajectory of early-phase research in Italy. In parallel, a dedicated comparative analysis across EU Member States is warranted to evaluate national capacities, identify best practices, and assess the impact of ACT EU (Questions and answers on the european biotech act, 2026) and other harmonisation initiatives on the competitiveness of early-phase research across Europe.

## Data Availability

Publicly available datasets were analyzed in this study. This data can be found here: Data partially available online as open-source information on the official AIFA website and on the open-access platforms EudraCT and CTIS: OsSC (https://www.aifa.gov.it/sperimentazioni-cliniche) and EudraCT (https://www.clinicaltrialsregister.eu/ctr-search/search) for CTAs submitted under Directive 2001/20/EC, covering the period 01 January 2016–30 January 2023. CTIS (https://euclinicaltrials.eu/search-for-clinical-trials/?lang=en) for CTAs submitted under Regulation (EU) No. 536/2014, active from 31 January 2022 onward.

## Footnotes

1 Italian public or private research hospitals recognized for their dual mission of providing specialized healthcare and conducting high-level biomedical research.

2 For the purpose of review, all Phase I–related categories were grouped under Phase I. Phase I Categories: Phase I - First administration to humans; Phase I–Other; Phase I - Bioequivalence Study; Phase I/II - First administration to humans; Phase I/II - Other; Phase I/II/III; Phase I/III.
